# The Development of a Sensitive and Selective Method for the Quantitative Detection of Ricin via ICP-MS Combined with Metal Element Chelated Tag and Modified Nanoparticles

**DOI:** 10.3390/ijms26125641

**Published:** 2025-06-12

**Authors:** Long Yan, Kexuan Li, Jina Wu, Zhongfang Xing, Xiaosen Li, Shilei Liu

**Affiliations:** State Key Laboratory of Chemistry for NBC Hazards Protection, Beijing 102205, China; yanl_allen@163.com (L.Y.); lkxlikexuan@outlook.com (K.L.); wujina09@163.com (J.W.); 18518417622@163.com (Z.X.)

**Keywords:** ricin, inductively coupled plasma mass spectrometry, metal element label, quantitative detection

## Abstract

As a type II ribosome-inactivating protein (RIP-II) toxin, Ricin has garnered widespread recognition due to its inherent qualities as an easily prepared and highly stable substance, posing serious implications as a potential chemical and biological terrorist threat. For the detection of ricin, traditional immunoassay technologies, including methods like peptide cleavage combined with liquid chromatography mass spectrometry (LC-MS) or the more commonly used enzyme-linked immunosorbent assay (ELISA), have offered reliable results. However, these techniques are unfortunately limited by the requirement of a complex sample pretreatment process, which can be time-consuming and labor-intensive. In an effort to overcome these limitations, a highly sensitive and selective method was introduced via metal element labeling combined with inductively coupled plasma mass spectrometry (ICP-MS) in this research. The method centered on designing and synthesizing a europium-labeled compound (DOTA-NHS-Eu) that specifically targets the amino groups (-NH_2_) on ricin. The compound, coupled with the application of specific magnetic beads, achieved the specific enrichment and subsequent quantitative detection of ricin by ICP-MS, which is based on the amount of europium element present. The established method demonstrated high specificity for ricin recognition, with a signal response to bovine serum protein that was found to be less than 10% of that for ricin. Furthermore, the calibration curve created for the method (y = 81.543x + 674.02 (R^2^ > 0.99)) for quantifying ricin in a concentration range of 1.0–100 μg/mL demonstrated good linearity. The method was further evidenced by the limit of detection and quantitation results of 0.1 and 1.89 μg/mL, respectively. Collectively, these findings suggested that the research has offered a highly sensitive and selective method for ricin detection, which was not only easy to operate but also provided efficient results. The scheme showed great potential for the verification of chemical weapons and the destruction of toxic chemicals, therefore representing a significant advancement in the field of biomolecular detection and analysis.

## 1. Introduction

**Figure 1:**
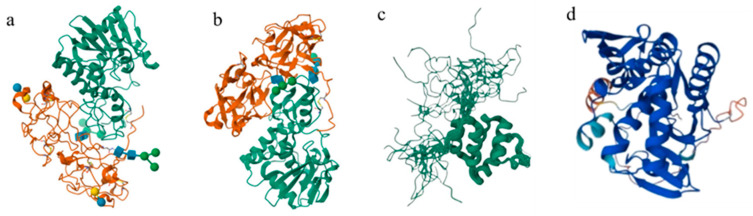
The structure of different phytotoxic proteins: (**a**) ricin; (**b**) abrin; (**c**) crotin; (**d**) curcin.

Biotoxins are recognized as biochemical substances derived from biological organisms that exhibit toxic effects on other biological species [1,2]. They are notable for diversity and complexity and can be divided into plant toxins, animal toxins, and microbial toxins according to their sources [1,2,3]. Compared to toxins from animal or microbial sources, plant toxins have garnered increasing attention in recent years due to their ease of preparation, wide availability, good stability, and potential use as bioterrorism agents. Plant toxins include terpenoids, phenolic compounds, alkaloids, polypeptides, and proteins such as ricin. Most plant proteins contain a single polypeptide chain similar to the protein chain of ricin and are called ribosome-inactivating proteins (RIPs); this category includes Abrin, Crotin, and Curcin [4,5] (Figure 1). Ricin can be categorized as one of the type II ribosome-inactivating proteins (RIP-II), which represents an important class of protein toxins consisting of A and B chains connected by an interchain disulfide bond. The B chain possesses galactose-binding characteristics that can specifically bind to the receptor on the surface of eukaryotic cell membranes. Meanwhile, the A chain with N-glycosidase activity is easily introduced into cells. Depurination occurs with specific ribosome sites to inactivate ribosomes, and cytotoxicity has been exhibited by inhibiting protein synthesis. Ricin has been confirmed to be one of the most toxic plant toxins for cell lines with IC50s (concentration inhibiting protein synthesis by 50%) ranging from less than 0.1 to 1 pM [6,7]. Oral administration of ricin has a low lethal dose (LD) for 50% of animals (LD50s) of 20 to 30 mg/kg in rats and 15 to 35 mg/kg in mice; these values are even lower for intravenous injection [8,9,10,11]. As the only protein toxin, ricin has been classified in the prohibition list of the Chemical Weapons Convention (CWC) [12]. Consequently, the development of rapid and accurate identification methods for ricin has already been a research focus in the field of toxins for several decades. The occurrence of ricin-related bioterrorism incidents in recent years has further promoted the development of accurate, sensitive, and rapid detection and identification technologies for type II RIPs. Due to the importance of type II RIP toxins, the Organisation for the Prohibition of Chemical Weapons (OPCW) has proposed technical requirements for the identification and analysis of relevant biotoxin samples [13].

The main detection and identification methods for highly toxic type II RIPs that have been established include immunological assays, qualitative or quantitative analysis via biological mass spectrometry, and toxin activity assessment based on depurination and cytotoxicity [14]. Immunological assays, which rely on antigen–antibody interactions and oligonucleotide aptamer-based recognition, have the advantages of high speed and sensitivity. As a widely utilized immunodetection method, the enzyme-linked immunosorbent assay (ELISA) has been extensively applied in detecting type II RIP toxins due to its versatility, high sensitivity, strong specificity, and ease of operation. For instance, the double antibody sandwich ELISA technique had been used for the quantitative detection of ricin in urine and serum samples, achieving a detection limit of 50 ng/mL [15]. In recent years, WB (western blotting) gradually became the predominant method for toxin detection. Lang et al. [16] developed the WB detection method for ricin using rabbit serum against RTA as the primary antibody, with a minimum detection limit of 1.0 ng. Delehanty et al. [17] achieved simultaneous detection of cholera toxin, staphylococcal enterotoxin B (SEB), ricin, and Bacillus within 15 min using an antibody microarray with charge-coupled elements, and the ricin detection limit was as low as 0.01 μg/mL. Additionally, oligonucleotide aptamer-based recognition methods also had been widely employed in type II RIP toxin detection. For instance, SELEX (Systematic Evolution of Ligands by Exponential Enrichment) technology has also been used to screen the appropriate aptamers for ricin and abrin, facilitating their quantitative detection with limits of quantification as low as 1.0 μg and 0.3 μg, respectively [18,19]. Furthermore, extensive methods have been developed on this issue, including fluorescence labeled peptide fragments [20], fluorescence resonance energy transfer [21,22], and immuno-sorbent surface area assays [22].

However, due to antigen–antibody interactions or oligonucleotide aptamer recognition, the traditional methods were easily generating false-positive results, particularly in complex samples. With the rapid advancement of biomass spectrometry, electrospray ionization (ESI) and matrix-assisted laser desorption/ionization (MALDI) have been widely applied for accurate protein identification [23,24,25]. These techniques not only provided precise molecular weight and structural sequence information but also enabled accurate quantification. For instance, Despeyroux et al. [26] confirmed the presence of ricin via ESI quadrupole (Q) mass spectrometry, obtaining an average relative molecular mass of 63 kDa, corresponding to the known molecular mass of ricin. Moreover, given the highly toxic nature of type II RIP toxins, their identification could be achieved through activity analysis. Activity analysis of type II RIP toxins primarily included depurination reaction assays based on N-glycosidase activity and cytotoxicity assays [24]. Both methods offered simple and sensitive means for detecting toxin toxicity, serving as effective supplements to the existing detection methods. Despite the development of multiple detection approaches, current techniques still exhibit limitations. For example, while activity-based identification could confirm the presence of a toxin, it failed to determine the structure or type. Additionally, employing MALDI-TOF required complex sample pretreatment, which was time-consuming and limited its practical application [25]. The rapid and accurate identification and detection of type II RIP toxins had become a critical focus in anti-bioterrorism development planning worldwide due to their potent toxicity, ease of preparation, and difficulty of prevention. Samples containing type II RIP toxins often possessed complex matrices, significantly increasing the challenges for detection and identification [27]. Therefore, the development of specific identification and accurate quantitative detection techniques for biotoxins in complex matrices, such as food, environmental, and biological samples, held substantial practical and military importance in fields of protection from chemical and biological warfare agents, chemical weapon verification, and anti-terrorism. This remained a key research focus for unequivocal biotoxin identification in the future.

The metal element chelated tag (MECT) had gained widespread application in the field of biomarker analysis in conjunction with mass spectrometry due to the excellent stability, cost-effective labeling reagents, and straightforward operational process. These qualities supplied the technical demands of protein quantification [28]. The method of MECT extended across various mass spectrometry platforms, encompassing ionization techniques like ESI-MS and MALDI-TOF-MS, as well as inorganic mass spectrometry, notably inductively coupled plasma mass spectrometry (ICP-MS). Its capacity to precisely quantify proteins or polypeptides had increasingly demonstrated its advantages in protein quantitative research. ICP-MS offered significant benefits in the determination of metal elements [29]. The low detection limit, a wide linear range, and the ability to simultaneously detect multiple elements allowed its widespread application in the analysis of most metal elements. The method had also been widely applied in antigen–antibody immunoassays in the past decades [30]. By labeling antibodies or antigens with metal elements and exploiting the specific noncovalent binding between them, metal elements could be indirectly tagged to the antigen or antibody of interest. The approach eliminated the need for complex chromatographic separation techniques, instead leveraging the high sensitivity and accuracy of ICP-MS in simultaneously quantifying multiple metal elements to achieve antigen quantification [31,32]. The immunoassay technology utilizing ICP-MS based on metal element labeling exhibited clear advantages, including high sensitivity, precision, and accuracy [33,34]. In this study, we aimed to develop a trace analysis method for ricin that combines metal element labeling with immunity-enrichment and ICP-MS technology. The specific detection technique was successfully established for ricin, offering an alternative method for the specific identification and accurate quantification of ricin in various complex matrices.

## 2. Results and Discussion

### 2.1. The Design Principle and Characterization Analysis for Element Label Tags

**Figure 2:**
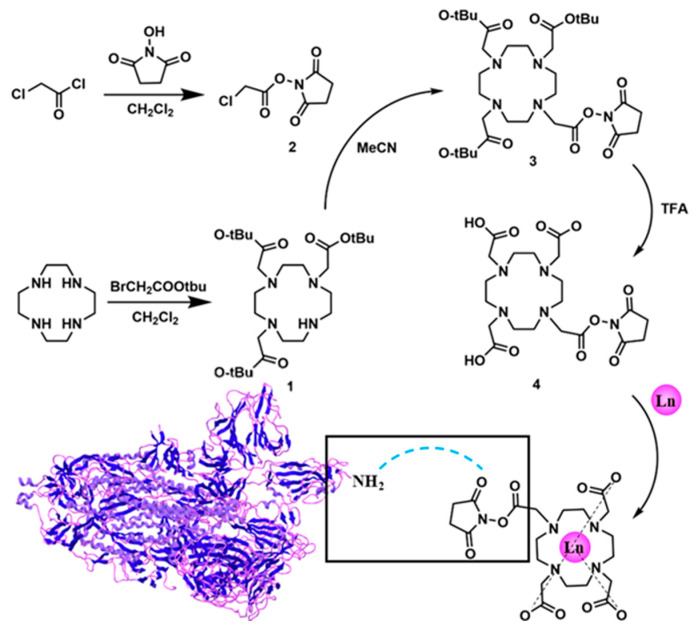
The design principle for the element label tag.

The ionization efficiency of lanthanide europium (Eu) in inductively coupled plasma mass spectrometry (ICP-MS) was remarkably high, with its atomic mass surpassing 100, thus exhibiting minimal interference from polyatomic ions and a low biological background [35,36]. Leveraging this advantage, we synthesized the 1,4,7,10-tetraazacyclododecane-1,4,7,10-tetra-acetic acid chelating molecule (DOTA) that demonstrated a strong coordination binding affinity for Eu ions (the synthesis and NMR characterization of the products of each step and the final DOTA-NHS-ester have been presented in the Supporting Information, Figures S1–S6). Firstly, DOTA-Eu complexes could be readily formed through the establishment of stable coordination bonds between N and O atoms and Eu3+ ions, with the stability constant logK of these complexes ranging from 22 to 26 [37]. On the other hand, targeting the amino group (-NH2) present in ricin molecules, the side chain of DOTA was modified with an N-hydroxy succinimide (NHS) ester functional group. This NHS-ester group was highly reactive towards amino groups [38], forming a stable amide covalent bond under mild conditions (at room temperature and within a pH range of 7.0 to 8.5). Consequently, ricin or other proteins with exposed amino groups could be efficiently labeled with the DOTA-NHS-Eu elemental tag (shown in Figure 2).

### 2.2. Optimization of the Synthesis Scheme and Characterization of DOTA-NHS-ester

**Figure 3:**
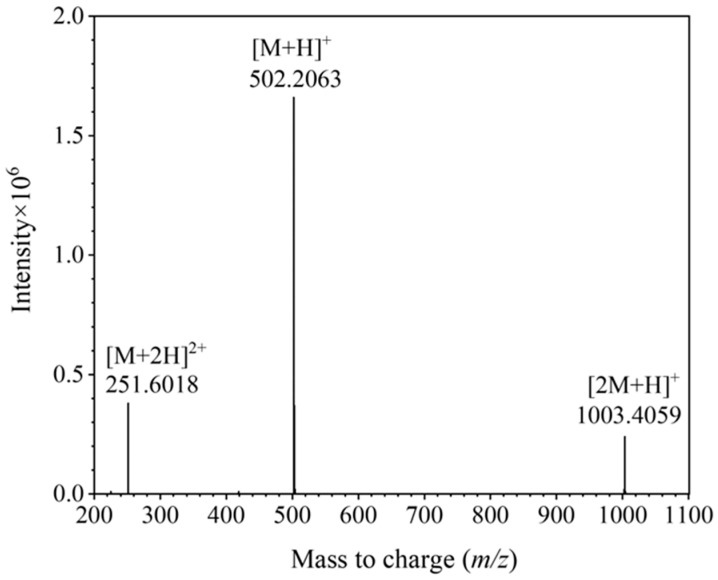
The detected mass-to-charge for DOTA-NHS-ester via mass spectrometry.

DOTA-NHS-ester was synthesized starting from 1,4,7,10-tetracyclododecane (Cyclen) via the detailed synthetic pathway presented in Figure 2. Initially, a selective modification was carried out on three of the secondary amine groups of Cyclen, which were substituted with tert-butyl acetate moieties. The process was achieved through a substitution reaction involving tert-butyl bromoacetate and the secondary amines, yielding the intermediate DO_3_AtBu (**1**). The details of the synthesis scheme are shown in the Supporting Information (SI). According to the established synthesis scheme, DOTA-NHS-ester was successfully synthesized and the theoretical molecular mass was 501.2071 (the molecular formula was C_20_H_31_N_5_O_10_). The detected molecular mass-to-charge ratio (*m*/*z*) established via ESI-Q-TOF mass spectrometry, which is shown in Figure 3, found [M + H]^+^ 502.2063, [M + 2H]^2+^ 251.6018, and [2M + H]^+^ 1003.4059.

**Figure 4:**
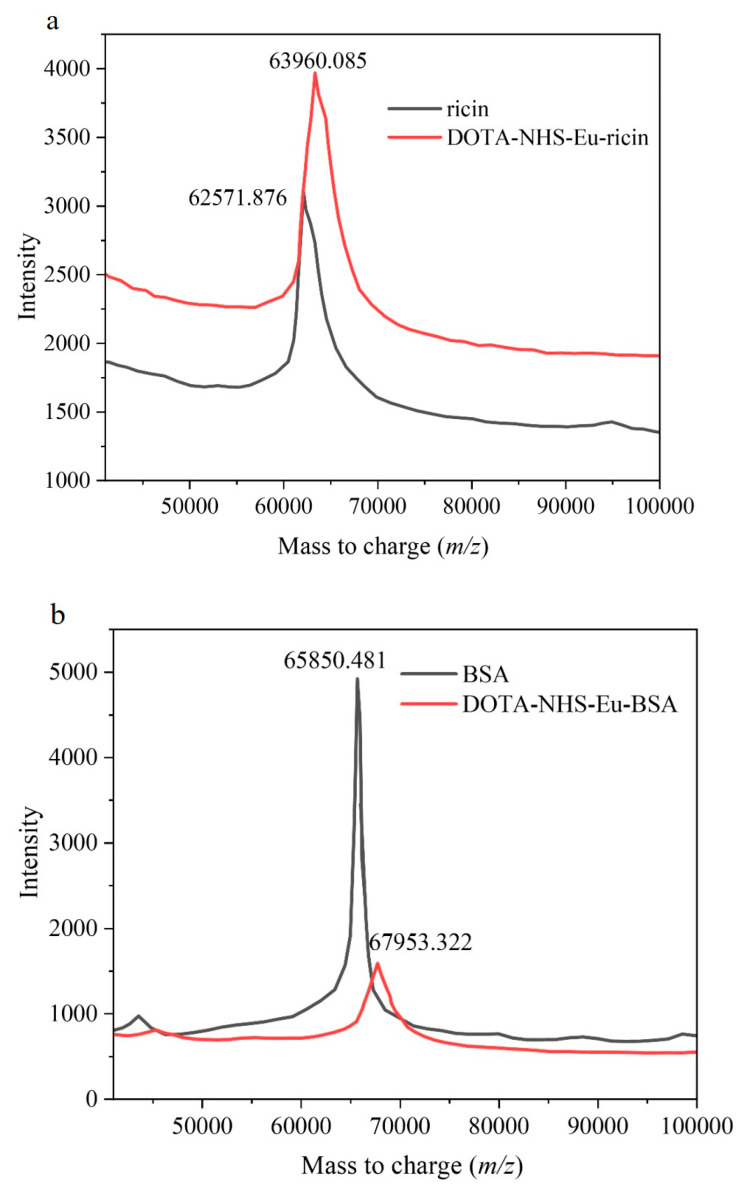
The results of the labeling efficiency for DOTA-NHS-Eu with Ricin and BSA. (**a**). The labeling results for ricin; (**b**) the labeling results for BSA.

To investigate the conjugation efficiency of the synthesized DOTA-NHS-Eu with specific proteins, Ricin and BSA were labeled with DOTA-NHS-ester complexed with Eu^3+^. An amount of 500 μL of ricin aqueous solution and BSA aqueous solution with the same concentration of 200 mg/L were prepared. Subsequently, 100 μL of DOTA-NHS-Eu aqueous solution (1 mg/mL) was added to each sample, followed by the addition of 400 μL of a 200 mM NaHCO_3_ aqueous solution (pH = 8.0). The reaction was conducted at 28 °C for 3 h. After the incubation was terminated, the labeled protein conjugate was purified using an ultrafiltration centrifuge tube with a molecular weight cutoff of 10 kDa. The resulting concentrated solution was then diluted to 1 mL with a Tris-HCl buffer solution (100 mM, pH 7.4, containing 0.05% Tween 20). The labeling efficiency was evaluated by MALDI-TOF-MS analysis and the results are presented in Figure 4, revealing that each ricin molecule was labeled with two DOTA-NHS-Eu molecules, while each BSA molecule was labeled with three DOTA-NHS-Eu molecules.

### 2.3. The Design Principle and Preparation Process for the Specifically Ricin-Enriched Magnetic Beads

**Figure 5:**
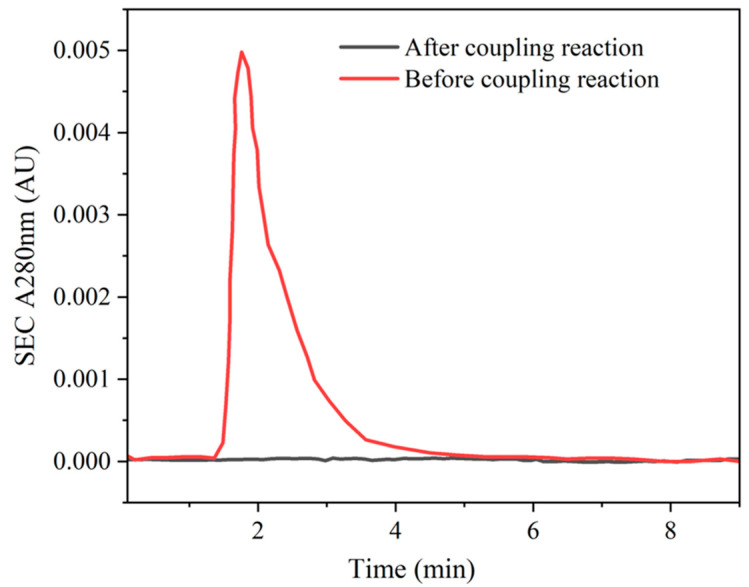
The coupling efficiency for antibodies and magnetic particles.

The preparation of ricin-specific enrichment magnetic beads mainly involved modifying ricin antibodies on the surface of magnetic beads through the reaction between NHS (N-hydroxysuccinimide) on the surface of the magnetic beads and the amino groups on the ricin antibodies, and then specifically enriching ricin in aqueous solutions through immune reactions and magnetic separation. In this study, NHS-activated magnetic agarose beads were conjugated with ricin antibodies. Following pretreatment with washing buffer and coupling buffer, the beads were subjected to incubation with the ricin antibody solution. Subsequently, the antibody-modified magnetic particles were isolated via magnetic separation. The amino groups present on the ricin antibodies underwent the reaction with the NHS ester groups on the magnetic beads, resulting in the formation of stable amide covalent bonds. To assess the coupling efficiency, the supernatant obtained post-conjugation was collected and subjected to analysis using size-exclusion chromatography (SEC) via gel filtration. The absorbance at A280 nm, which corresponds to the characteristic absorption wavelength of proteins, was measured. As illustrated in Figure 5, no residual antibodies could be detected in the supernatant following conjugation, thereby confirming the successful coupling of antibodies to the magnetic beads.

**Figure 6:**
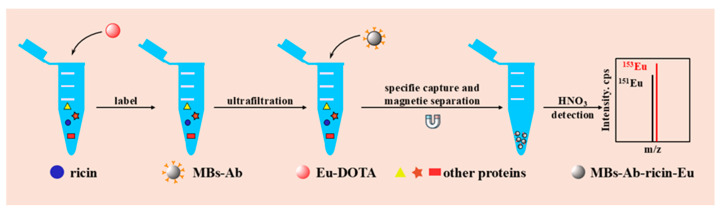
The methodology for the detection of ricin via ICP-MS combined with ricin-enriched magnetic beads.

The ricin monoclonal antibody was successfully immobilized onto the surface of magnetic beads, facilitating the detection of the ricin concentration in the sample through specific antigen–antibody recognition. Concurrently, ricin molecules in the sample were effectively labeled with elemental tags, which were subsequently released and quantified via inductively coupled plasma mass spectrometry (ICP-MS) analysis. Initially, an excess of elemental labels was supplied to the sample to ensure that all amino-group-containing molecules reacted with DOTA-NHS-Eu. Subsequently, leveraging the antibody-modified magnetic beads, DOTA-NHS-Eu-labeled ricin in the sample was specifically enriched, while other molecules such as proteins could be efficiently segregated. Finally, 2% nitric acid was employed to release europium (Eu) bound to ricin. The Eu signal was then detected by ICP-MS, enabling quantitative detection of ricin in the sample. The detection methodology is illustrated in Figure 6.

### 2.4. The Verification of Specificity and Sensitivity for the Sample Preparation via Ricin-Enriched Magnetic Beads

**Figure 7:**
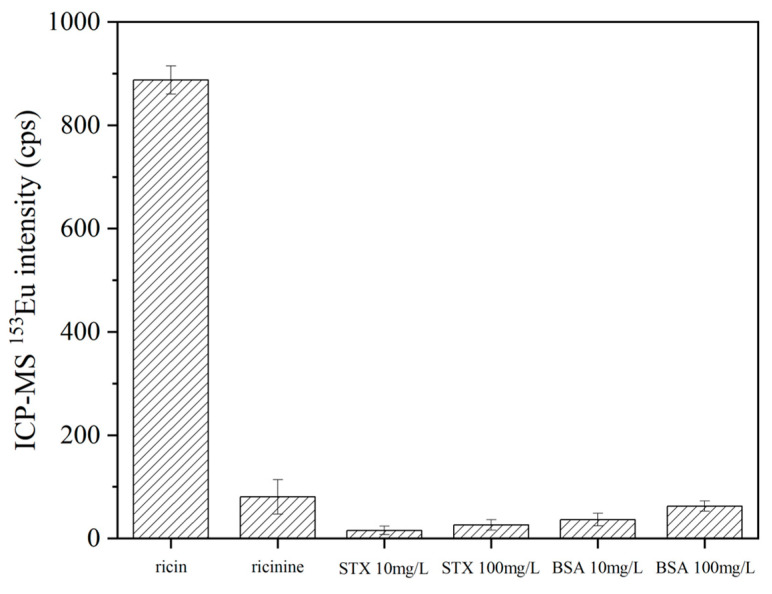
The specificity for the ricin-enriched magnetic beads.

To validate the specificity and selectivity of the established sample preparation, labeling experiments were performed utilizing DOTA-NHS-Eu element tags on ricin, ricinine, bovine serum albumin (BSA), and saxitoxin (STX). Specifically, 1 μL aliquots of 10 mg/L aqueous solutions of ricin and ricinine and 10 mg/L and 100 mg/L of BSA and STX aqueous solutions were prepared, respectively. To each solution, 20 μL of immune reaction buffer and 2.5 μL of ricin antibody-modified specific enrichment magnetic beads were introduced, followed by incubation at 37 °C for 1 h. After the reaction, the magnetic beads underwent sequential washing with 200 μL of immune reaction buffer and deionized water twice. Subsequently, 50 μL of 2% HNO_3_ was added for elution and the mixture was allowed to react at 25 °C for 3 h under suspension conditions. The supernatant was then collected via magnetic separation. The magnetic beads were further washed twice with 50 μL of 2% HNO_3_, and the washing solutions were collected with the same procedure. The supernatants obtained from the aforementioned three steps were combined and diluted to a final volume of 500 μL for ICP-MS analysis. The experiment was repeated and analyzed independently (*n* = 6) the same day. As depicted in Figure 7, the results revealed that the signal responses of ricinine, BSA, and STX were all below 10% of the ricin response, thereby demonstrating that the specifically prepared ricin enrichment magnetic beads exhibited exceptional selectivity.

### 2.5. Linear Range, Limit of Detection (LOD), and Limit of Quantitation (LOQ)

**Figure 8:**
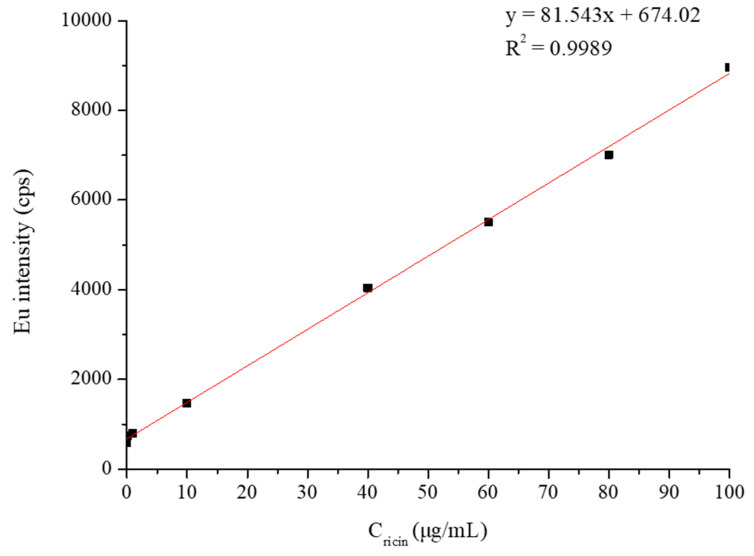
The standard working curve of ricin aqueous solution detection.

The signal responses for ricin in the solution samples via the established method that combined specific enrichment magnetic beads with ICP-MS detection exhibited an excellent linear relationship in the range from 0.1 to 100 μg/mL (shown in Figure 8), while the standard working curve of ricin was y = 81.543x + 674.02 (R^2^ = 0.9989). The increase in the signal showed a smaller effect than that calculated from linear regression when the spiked concentration was beyond 100 μg/mL. Take the sample with the spiked concentration of 200 μg/mL as an example: the response signal was approximately 12,500, which was significantly lower than the theoretical calculated value of 17,000 according to the standard curve (the fitting result for the standard curve including the concentration of 200 μg/mL is shown in the Supporting Information, Figure S7). According to the signal value of Eu in ICP-MS at an injection flow rate of 300 μL/min and the calculated equation, the limit of detection (LOD) of this method for ricin aqueous solution was calculated to be 0.1 μg/mL, and the limit of quantitation (LOQ) for ricin aqueous solution was 1.89 μg/mL.

The accuracy and precision of intraday and interday analyses were measured for the QCL, QCM, and QCH samples. The intraday accuracies and RSD (*n* = 6) for ricin ranged between 97.5 and 106.2% with precision values of ≤4.93%. The interday accuracies and RSD (*n* = 6) for ricin were between 96.7 and 98.2% with precision values of ≤4.88 (shown in Table 1). The accuracy and precision results indicated that our established method could satisfy the FDA’s guidance for bioanalytical method validation, which showed great potential for the analysis of clinical samples and CWC verification.

### 2.6. The Analysis Results for the Actual Environmental Samples

The actual environmental samples, including the soil samples and river water samples, were collected from the local area. The pretreated samples (shown in Section 3.3) were concentrated to a final volume of 50 μL using a 10 kDa ultrafiltration centrifuge tube under the conditions of 25 °C, 7500× *g*, and a duration of 15 min. Subsequently, the samples were diluted to 500 μL with a 0.2 M NaHCO_3_ buffer solution (pH = 8.0) supplemented with 0.05% Tween 20. Following the ultrafiltration centrifugation, 100 μL of the 1.0 mg/mL DOTA-NHS-Eu aqueous solution was introduced into the sample solution. Upon terminating the reaction, the sample was ultrafiltered again via a 10 kDa ultrafiltration centrifuge tube to eliminate the excess probe (ricin was kept due to the molecular weight of 66 kDa). The concentrated solution was then diluted to 1 mL with a 0.1 M Tris-HCl buffer solution.

Prior to utilization, magnetic beads were subjected to three washing cycles with the immune reaction solution and subsequently dispersed in an equivalent volume of the same solution. Then, 1 μL of the prepared environmental sample solution was transferred to the centrifuge tube, followed by the addition of 20 μL of the immune reaction solution and 2.5 μL of specific ricin enrichment magnetic beads. The mixture was incubated at 37 °C for 1 h. Following the reaction, the magnetic beads were washed three times with 200 μL of the immune reaction solution and once with deionized water. Subsequently, 50 μL of 2% HNO_3_ was added to the washed magnetic beads, which were maintained in suspension at 25 °C for 3 h. The supernatant was separated and collected. The magnetic beads were further washed twice with 2% HNO_3_, and the resulting supernatants were also collected. Finally, all supernatants were combined, thoroughly mixed, and diluted to 500 μL for ICP-MS analysis.

**Figure 9:**
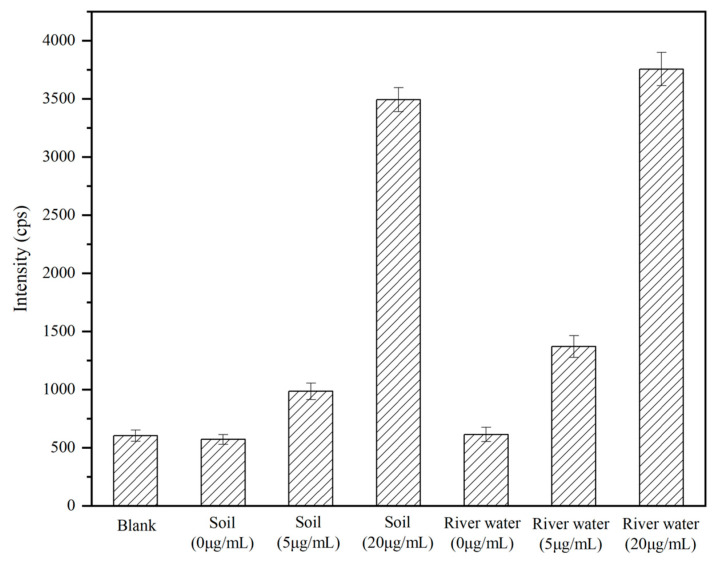
The analysis results for the actual environmental samples.

The established method in this study was employed for the analysis of actual samples. Simulated contaminated environmental samples were utilized to quantitatively detect and analyze ricin added to various matrices, including soil and river water. The results are illustrated in Figure 9. Notably, the blank samples of soil and river water demonstrated background levels akin to the baseline of the instrument. Both the 5 mg/L and 20 mg/L spiked environmental samples were successfully identified. These findings substantiated the applicability of this method for analyzing ricin in actual environmental samples.

### 2.7. The Assessment of Matrix Effects

Matrix effects (ME%) could result in the inhibition or enhancement of the analyte signal due to the matrix solution. The accepted and appropriate criteria were applied for the evaluation of the ME% based on the following equation.

$$\mathrm{ME}(\%)=\left|{\mathrm{Singal}}_{\mathrm{solvent}}-{\mathrm{Singal}}_{\mathrm{matrix}}\right|/{\mathrm{Singal}}_{\mathrm{solvent}}\times 100\%$$

The signal responses of the ricin in environmental samples (Singal_matrix_) were reduced by the signal responses of the target in the deionized water (Singal_solvent_) and then divided by Singal_solvent_. ME values were categorized as mild (0–20%), moderate (20–50%), and significant (>50%) [39]. The ME (%) was calculated with the spiked concentration being 5.0 mg/L for ricin in the soil and river water compared to deionized water. The values were 17.3 ± 1.2% and 12.5 ± 1.7%, respectively. The results indicated that the method developed in this study effectively eliminated the matrix effect and exhibited superior anti-interference capability.

## 3. Materials and Methods

### 3.1. Safety Precaution

As the highly toxic protein is an RIP toxin and could infect humans via ingestion or exposure through broken skin, all the ricin-related experiments should be performed in a biosafety level 2 cabinet equipped with a HEPA filter. Ricin-contaminated solutions and consumables must be decontaminated with bleach solution. All relevant operations should be carried out by professional laboratory operators.

### 3.2. Chemicals and Instruments

1,4,7,10-tetraazacyclododecane-1,4,7,10-tetraacetic acid (DOTA)-N-hydroxysuccinimide (NHS)-Eu (DOTA-NHS-Eu) was synthesized in-house and the purities were verified at ≥95% by 600 MHz nuclear magnetic resonance (NMR) [Bruker BioSpin NMR CP BBO 600S3 (600.13 MHz), Bruker, Karlsruhe, Germany]. Europium (III) chloride hexahydrate (EuCl_3_·6H_2_O, 99.99%) was purchased from Sigma-Aldrich (Saint Louis, MO, USA). The magnetic nanoparticle MNP (NHS-activated Magarose Beads) was purchased from Smart-Lifesciences Co. (Changzhou, China). The anti-ricin polyclonal antibody (pAb) was synthesized in-house. The centrifugal filter device with a molecular weight cutoff of 10 kDa was purchased from Merck Millipore (Darmstadt, Germany). Deionized water was purchased from Watsons Water. All the reagents and materials were of analytical grade or higher.

The related buffers were prepared in accordance with the following composition:

(1) The coating buffer was Tris-HCl buffer solution (100 mM, pH 7.4) containing 0.05% polysorbate 20.
(2) The cleaning solutions were 1 mM HCl, 0.1 M acetic acid-sodium acetate, 0.5 M NaCl, pH 3.0, and 0.1 M Tris-HCl, 0.5 M NaCl, pH 8.0.
(3) The protective solution was 1 × PBS containing 20% ethanol.
(4) The immune reaction solution was 0.1 M Tris-HCl, 0.05% tween 20, pH 7.4.
(5) The coupling solution was 0.2 M NaHCO_3_, 0.5 M NaCl, pH 8.0.
(6) The blocking solution was 0.1 M Tris, pH 8.5.

The equipment used for sample preparation included a rotary evaporator (Eppendorf, Concentrator Plus, Hamburg, Germany) and a high-speed centrifuge (Sigma, 3-30KS, Darmstadt, Germany). The prepared samples were analyzed by an ICAP Q ICP-MS (Thermo Scientific, Waltham, MA, USA).

### 3.3. Sample Preparation

#### 3.3.1. Synthesis and Characterization of DOTA-NHS-Eu

The synthesis of DOTA-NHS could be successfully achieved starting from 1,4,7,10-tetracyclododecane (Cyclen), as illustrated in Figure 2. In the initial step, three of the secondary amine sites on Cyclen underwent the substitution reaction with tert-butyl bromoacetate, resulting in the formation of DO_3_AtBu (**1**) where these sites were modified with tert-butyl acetate groups. Subsequently, ClCH_2_CONHS-ester (**2**) could be synthesized through the reaction between chloroacetyl chloride and N-hydroxysuccinimide. This compound then reacted with the remaining secondary amine site on DO_3_AtBu (**1**), leading to the formation of DO_3_AtBu-NHS-ester (**3**). In the third step, the three tert-butyl protecting groups on compound **3** were removed using trifluoroacetic acid (TFA), yielding DOTA-NHS-ester (**4**). Lastly, under neutral conditions, compound **4** could form a stable coordination complex, DOTA-NHS-Eu, with Eu^3+^. The reaction system contained 1 mg/mL DOTA-NHS-ester solution and 1 μL of 500 mM EuCl_3_ at 28 °C for 1 h.

#### 3.3.2. Preparation of Specifically Ricin-Enriched Magnetic Beads

The solution of NHS activated Magarose Beads (0.6 mL, the volume of magnetic beads accounts for 20%) was washed three times with 1.0 mL of the cleaning solution and once with 1.0 mL of coupling solution. The washed magnetic beads were re-dissolved with 600 μL of coupling solution, and then 0.24 mL of ricin antibody aqueous solution of 3.58 mg/mL was added, resulting in a final concentration of 1.0 mg/mL. After incubation at 28 °C for 3 h, the supernatant was collected and the A = 280 nm was detected by size-exclusion chromatography to verify the coupling efficiency.

To prevent non-specific adsorption, the blocking solution and 6.0 mg of BSA were added to react for 1 h at 28 °C. The blocking liquid was removed and the magnetic beads were washed with cleaning solution and deionized water twice in sequence. The prepared beads were carefully stored in 0.6 mL protective solution at 2–8 °C for later use.

#### 3.3.3. Preparation of Selective Adsorption via DOTA-NHS-Eu and Ricin-Enriched Magnetic Beads

Firstly, 500 μL sample solution was mixed with 100 μL DOTA-NHS-Eu aqueous solution with a concentration of 1.0 mg/mL, and then 400 μL NaHCO_3_ aqueous solution with a concentration of 200 mM at a pH of 8.0 was added to react for 3 h at 28 °C. After the reaction, the sample combined with the DOTA-NHS-Eu tag was purified by ultrafiltration centrifuge tube with a cutoff capacity of 10 kDa. The concentrated solution was diluted to 1.0 mL with immune reaction solution at a concentration of 100 mM and pH = 7.4.

An amount of 1.00 μL of preconditioned sample solution was put into centrifuge tubes, and 2.50 μL of prepared ricin-enriched magnetic beads (0.86 mg/mL, MBs-Ab) solution was added. Then, 20.0 μL of the immune reaction solution was added to keep the magnetic beads in suspension at 37 °C for 1 h. The magnetic beads were washed three times with 200 μL immune reaction solution and then washed once with deionized water. A total of 50.0 μL of HNO3 with a concentration of 2% was added into the cleaned magnetic beads, which reacted at 25 °C for 3 h in suspension, then the supernatant was separated and collected. The magnetic beads were washed twice with 2% HNO_3_, and the supernatant was also collected. The supernatants obtained in this step were mixed and diluted to 500 μL for ICP-MS detection.

#### 3.3.4. ICP-MS Analysis

Argon gas with a high purity grade was applied for the reaction gas. The flow rates were as follows: 1.0 L/min for nebulizer gas, 0.8 L/min for auxiliary gas, and 14.0 L/min for cool gas. The optimized plasma power was 1550 W and the dwell time was set for 50 ms. Meanwhile, the focus lens voltage was 16.8 V and the peristaltic pump speed was up to 40 r/min. The injection flow rate was 300 μL/min.

#### 3.3.5. Method Verification and Calibration Curve

The calibration curve consisted of a blank sample and a seven-point calibration standard (0.1 μg/mL, 1.0 μg/mL, 10.0 μg/mL, 40.0 μg/mL, 60.0 μg/mL, 80.0 μg/mL, and 100.0 μg/mL) was fully validated. The limit of detection (LOD) was obtained according to the relationship between the blank signal and the slope of the calibration curve (the standard deviation of the blank signal divided by the slope of the calibration curve multiplied by ten). The lowest calibrator was utilized as the LOQ if the precision was lower than 20% and the accuracy ranged from 80 to 120%. The intraday precision and accuracy were measured with three QC samples for different concentrations of ricin (QCL for 1.0 μg/mL, QCM for 50.0 μg/mL, and QCH for 80.0 μg/mL), which were freshly prepared and analyzed independently (*n* = 6) the same day of use. No less than six measurements were completed within one week for the evaluation of precision and interday accuracy.

#### 3.3.6. Actual Sample Analysis

Actual soil samples and river water samples (from Jingmi Water Diversion Channel) were collected from the local area. Ricin toxin solution was added to prepare the spiked actual water samples with final concentrations of 5 mg/L and 20 mg/L. Both the spiked actual water samples and the blank water samples were centrifuged at 4 °C and 15,000 r/min for 20 min to remove solid insoluble substances. For the sample preparation of soil samples, the phosphate buffer solutions containeing ricin (final concentrations of 5.0 mg/L and 20 mg/L) were spiked. Both the spiked soil samples and the blank soil samples were also centrifuged at 4 °C and 15,000 r/min for 20 min to remove solid insoluble substances. The supernatants of the pretreatment actual samples were then collected for evaluation.

## 4. Conclusions

Based on the structural features of ricin and the intrinsic advantages of inductively coupled plasma mass spectrometry (ICP-MS), our research designed and synthesized an elemental-labeled probe, DOTA-NHS-Eu, which specifically targeted the amino group (-NH_2_) of the ricin molecule. Ricin-specific magnetic beads were utilized for the quantitative determination of ricin via ICP-MS, demonstrating exceptional selectivity and sensitivity in detecting and quantifying the toxin. The standard calibration curve expressed as y = 81.543x + 674.02 (R^2^ > 0.99) was established within the concentration range of 0.100–100 μg/mL, confirming the excellent linear relationship. Leveraging the high detection sensitivity of ICP-MS for europium (Eu), the method exhibited excellent limits of detection (LODs) and limits of quantitation (LOQs). In summary, this approach offers superior selectivity and sensitivity for ricin detection and can be extended to other ribosome-inactivating protein type II (RIP-II) toxins, such as abrin. Furthermore, it showed potential for detecting specific protein toxins in complex matrices. The sample pretreatment method established in our research primarily focused on the preparation of antibody-conjugated magnetic beads, which could be stored for subsequent use. Compared to the ELISA operation, which was labor-intensive and demanded greater technical expertise for operators, the established method was easily operated and facilitated high-throughput simultaneous analysis, positioning it as a promising technique for ricin analysis. Additionally, the developed method via ICP-MS detection obtained superior qualitative capabilities and specificity compared to ELISA, which was mainly based on spectrophotometric detection.

More importantly, the designated laboratory had attained the official designation from the OPCW and had the ability for the analysis of authentic environmental and biomedical samples exposed to chemical weapons. As mandated by OPCW guidelines, the analytical methodologies developed must possess the capability to detect chemical warfare agents or the corresponding markers at concentrations as low as 1.0 μg/mL in environmental matrices and 1.0 μg/mL in biomedical samples. The methodology established within this study not only fit the stringent official standards but also demonstrated considerable promise for practical application in chemical weapons verification protocols. Our research developed a toxin analysis method via antibody-conjugated magnetic bead enrichment, metal ion modification, and ICP-MS detection. The approach was also applicable to the analysis and detection of other protein-based toxins. Future work will investigate the research in abrin or other type II ribosome-inactivating protein (RIP-II) toxins.

## Figures and Tables

**Table 1:** The standard curve of ricin with the R^2^, accuracy, and precision of the QC samples.

Analytes	Linear Range (μg/mL)	Linear Equation			R^2^		
Ricin	0.1–100	y = 81.543x + 674.02			0.9989		
Sample ID	theoretical concentration (μg/mL)	intraday (*n* = 6)			interday (*n* = 6)		
		calculated concentration (μg/mL)	Accuracy (%)	RSD (%)	calculated concentration (μg/mL)	Accuracy (%)	RSD (%)
QCL	1.0	1.06 ± 0.01	106.2	4.93	0.98 ± 0.01	98.2	4.88
QCM	50.0	53.1 ± 1.1	106.1	3.66	48.57 ± 1.02	97.1	3.82
QCH	80.0	77.9 ± 1.2	97.5	2.89	77.4 ± 1.13	96.7	3.12

## Data Availability

Data is contained within the article and Supplementary Materials.

## Supplementary Materials

The following supporting information can be downloaded at: https://www.mdpi.com/article/10.3390/ijms26125641/s1.
