# Supplementary material for: The Development of a Sensitive and Selective Method for the Quantitative Detection of Ricin via ICP-MS Combined with Metal Element Chelated Tag and Modified Nanoparticles
Source: Int J Mol Sci. 2025 Jun 12;26(12):5641. doi: 10.3390/ijms26125641 (PMC12192896; doi:10.3390/ijms26125641)
Supplement: Supplementary file 1 [file ijms-26-05641-s001.zip › ijms-3641050-supplementary.pdf]

Supplementary Information

The development of sensitive and selective method for quantitative detection of ricin via ICP-MS combined with metal element chelated tag and modified nanoparticles

Long Yan, Kexuan Li, Jina Wu, Zhongfang Xing, Xiaosen Li*, Shilei Liu*

State Key Laboratory of Chemistry for NBC Hazards Protection, Beijing 102205, P. R. China

* Correspondence: momentday@126.com (Xiaosen Li); liu_shilei@lacricd.com (Shilei Liu)

The synthetic route for the DOTA-NHS-ester

1.1 Synthesis of DO_3AtBu (Compound 1 in the Figure.2, section 2.1)

Dichloromethane (DCM) solution of 1,4,7,10-tetraazacyclododecane (100 mM) was prepared under the mild nitrogen atmosphere. Triethylamine (5 eq) was added and the mixture was stirred in the ice bath for 15 min. Subsequently, the DCM solution of tert-butyl bromoacetate (3.25 eq, 1.6 M) was added dropwise. The reaction was maintained at 0 °C for 2 h and then allowed to warm to room temperature for 24 h. The mixture was washed three times with water, dried over anhydrous Na_2SO4 and filtered. The organic phase was removed under reduced pressure to yield the yellow oil. Further purification by silica gel column chromatography (ethyl acetate/methanol, 20:1, v/v) afforded the tri-tert-butyl ester-protected compound DO_3AtBu (the NMR results in Figure S1).

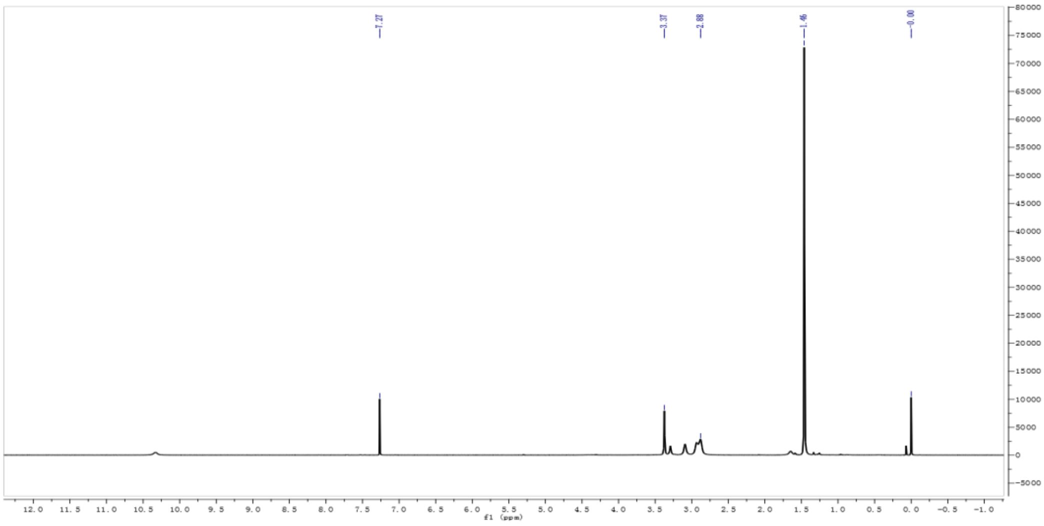

Figure S1 NMR results for the synthesis of compound 1 (δ: 4.37 (s, 2H), 2.87 (s, 4H))

1.2 Synthesis of $ClCH_2CONHS$-ester (Compound 2 in the Figure.2, section 2.1)

Chloroacetyl chloride (1 eq) was dissolved in DCM (50 mM), followed by the addition of triethylamine (1 eq) under ice-cooling. After stirring for 10 min, chloroacetyl chloride (1 eq) was slowly added. The reaction was stirred at 0 °C for 1 h and then at room temperature for 2 h. The organic layer was sequentially washed with ice-cold water, 50 mM HCl, 0.1 M $NaHCO_3$ and saturated NaCl in sequence. After drying over Na_2SO4 and filtration, the solvent was evaporated under reduced pressure to yield a white solid (the NMR results in Figure S2).

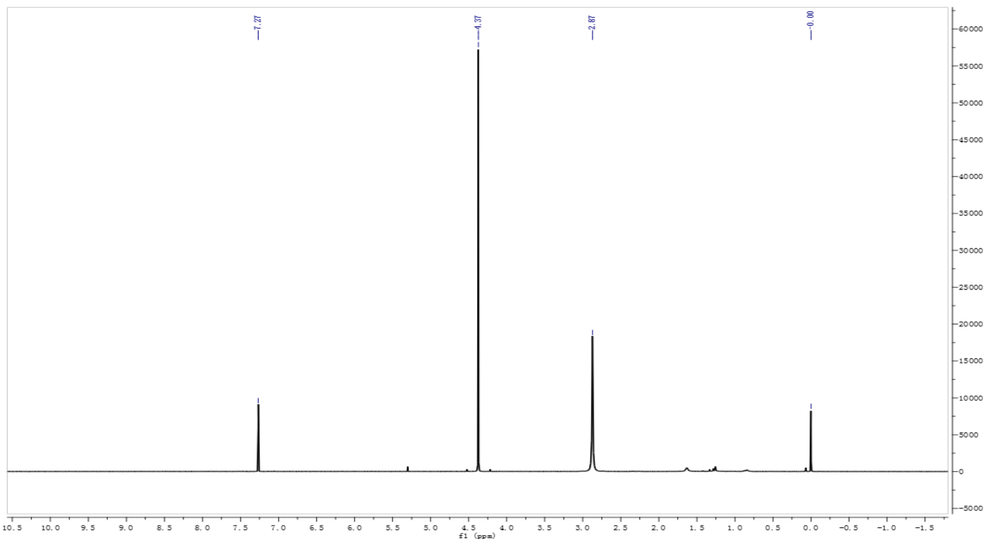

Figure S2 NMR results for the synthesis of compound 2 (δ: 4.37 (s, 2H), 2.87 (s, 4H))

1.3 Synthesis of DO3AtBu-NHS-ester (Compound 3 in the Figure.2, section 2.1)

Compound 1 (1 eq) was dissolved in acetonitrile, and K_2CO_3 (5 eq) was added. After stirring for 5 min at room temperature, Compound 2 (5 eq) was introduced. The reaction mixture was heated to 60 °C under reflux for 24 h. The precipitate was filtered off, and the solvent was removed under reduced pressure to afford a colorless oil. The step was optimized for reactant molar ratios (shown in the Figure S3) and reaction time (shown in the Figure S4). The NMR results was shown in Figure S5

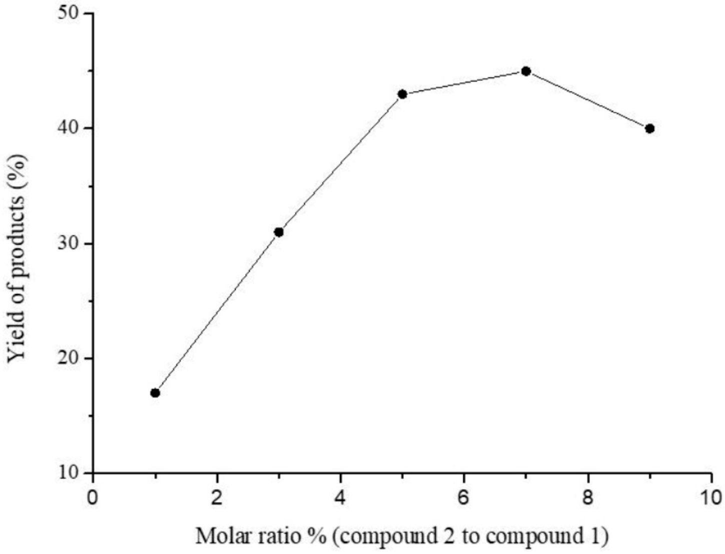

Figure S3 The optimized results for the molar ratio

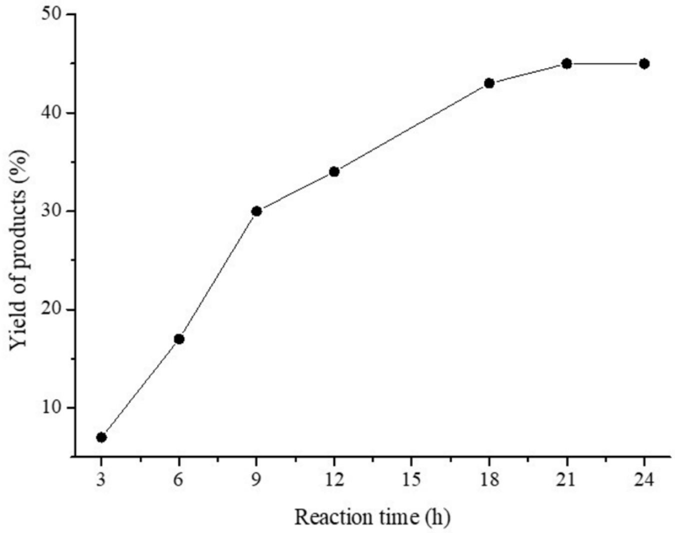

Figure S4 The optimized results for the reaction time

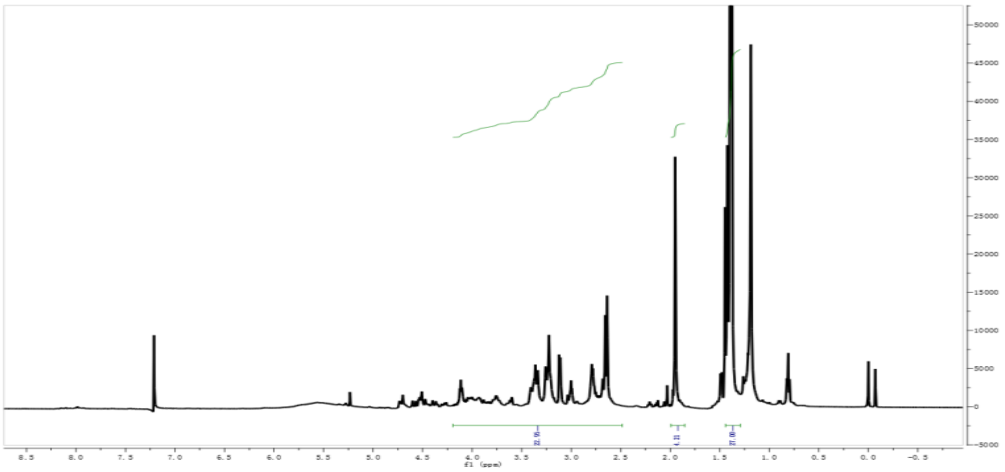

Figure S5 NMR results for the synthesis of compound 3 (δ: 3.49-2.90 (m, 18H), 2.87-2.55 (m, 10H), 1.40 (s, 27H))

1.4 Synthesis to DOTA-NHS-ester (Compound 4 in the Figure.2, section 2.1)

Compound 3 (1 eq) was treated with trifluoroacetic acid (TFA, 100 eq) at room temperature for 6 h to remove the tert-butyl ester groups. After repeated solvent evaporation under reduced pressure, the crude product was recrystallized using diethyl ether/methanol (40:1, v/v) to yield DOTA-NHS-ester (Compound 4) as a white solid (88% yield and the NMR results in Figure S6).

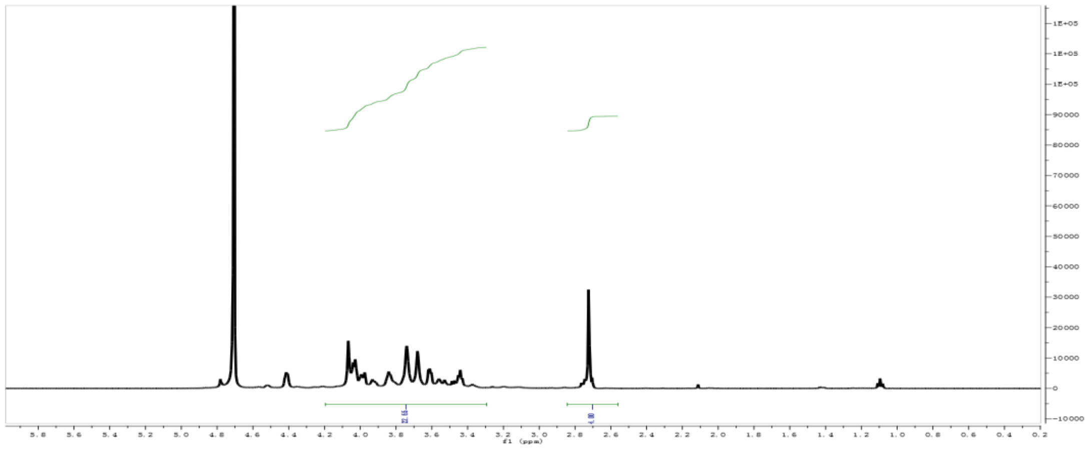

Figure S6 NMR results for the synthesis of compound 4 (δ: 4.47-3.43 (m, 24H), 2.72 (br, 4H))

The standard curve including the 200 μg/mL result

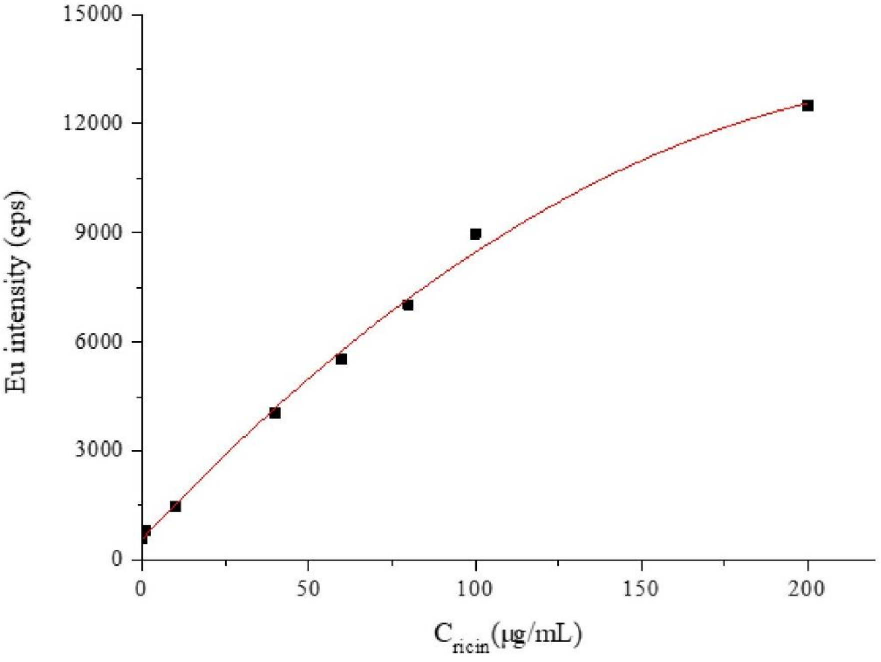

Figure S7 The fitting result for the standard curve including the concentration of 200 μg/mL
